# EP3 Receptor Deficiency Improves Vascular Remodeling and Cognitive Impairment in Cerebral Small Vessel Disease

**DOI:** 10.14336/AD.2021.0627

**Published:** 2022-02-01

**Authors:** Na Liu, Jie Tang, Yang Xue, Vincent Mok, Miaoyi Zhang, Xue Ren, Yilong Wang, Jianhui Fu

**Affiliations:** ^1^Department of Neurology, Huashan Hospital, Fudan University, Shanghai, China.; ^2^Gerald Choa Neuroscience Centre, Lui Che Woo Institute of Innovative Medicine, Division of Neurology, Department of Medicine and Therapeutics, The Chinese University of Hong Kong, Hong Kong, China; ^3^Department of Neurology, North Huashan hospital, Fudan University, No.108 Lu Xiang Road, Shanghai, China.; ^4^Department of Neurology, Beijing Tiantan Hospital, Capital Medical University, Beijing, China

**Keywords:** cerebral small vessel disease, hypertension, E-prostanoid receptor 3, transforming growth factor beta1, extracellular matrix

## Abstract

Aging and hypertension are major risk factors for cerebral small vessel disease (CSVD). Anti-hypertensive therapy has achieved effective; however, incomplete results in treating CSVD, suggesting the need for additional treatments. Targeting abnormal inflammatory responses has become a topic of research interest. Small artery remodeling is the main pathological feature of CSVD. Inhibition of the E-prostanoid 3 (EP3) receptor has been shown to attenuate vascular remodeling in peripheral organs; however, little is known about its role in CSVD. Therefore, we investigated whether the deletion of EP3 attenuates the development of CSVD in an animal model-- stroke-prone renovascular hypertensive rat (RHRsp). We found that the cerebral small arteries of RHRsp exhibited increased EP3 expression. Despite no alleviation of hypertension, the deletion of EP3 still attenuated the cerebral small artery remodeling of RHRsp, as evidenced by reduced overexpression of extracellular matrix (ECM) in the vessel. In vitro experiments indicated that EP3 deletion regulated the expression of ECM by downregulating TGF-β1/Smad signaling. Furthermore, the Morris water maze test and magnetic resonance test demonstrated that EP3 knockout attenuated cognitive impairment of the RHRsp, possibly through increased cerebral blood flow. Together, our results indicate that the deletion of EP3 attenuates vascular remodeling and vascular cognitive impairment induced by hypertension, and blockade of the EP3 receptor may be a promising strategy for the treatment of CSVD.

Cerebral small artery remodeling is the main pathological feature of arteriolosclerotic cerebral small vessel disease (CSVD), characterized by lumen narrowing and abnormal accumulation of extracellular matrix (ECM) in the vessel wall [1, 2]. Cerebral small artery remodeling reduces cerebral blood flow (CBF), leading to hypoperfusion of the brain [2, 3], which is closely related to the subsequent development of cognitive impairment [4, 5]. Hypertension is a major risk factor for CSVD [2], and chronic hypertension triggers vascular remodeling. However, recent clinical studies have also revealed that despite antihypertensive medications, some patients still present progressive vessel pathology and cognitive disorders [6, 7]. Given this, additional treatment, apart from anti-hypertension targeting vascular remodeling in CSVD, is becoming increasingly important.

Inflammation has long been listed as a candidate factor in the development of CSVD [8], and tackling abnormal inflammatory responses has become a potential treatment target for CSVD. One of the research focuses is on cyclooxygenase 2 (COX-2), a key enzyme in prostaglandin biosynthesis. A study showed that a *COX-2* genetic polymorphism in the Chinese population might contribute to the risk of developing white matter lesions [9]. Our previous study also revealed that using a COX-2 inhibitor can attenuate CSVD in an animal model [10]. However, chronic use of COX-2 inhibitors results in adverse side effects and increases the risk of vascular diseases, possibly by inhibiting the endothelial prostacyclin production [11], leading us to consider that modulating the prostaglandins and prostaglandin receptors downstream of the COX-2 pathway would be a much better strategy.

Among the prostaglandins synthesized from COX-2, prostaglandin E2 (PGE_2_) is the main product produced during inflammatory processes. PGE_2_ has four receptor subtypes, namely, E-prostanoid (EP) receptors (EP1-4) [12]. Elevated EP3 receptor expression has been reported in the small arteries of hypertensive animals [13], and inhibition of EP3 receptors has been shown to reverse cardiac hypertrophy induced by hypertension [14] or attenuated pulmonary arteriole remodeling in pulmonary hypertension [15]. However, little is known about the role of EP3 receptors in the vascular remodeling of CSVD induced by hypertension, and whether downregulation or blockade of EP3 will improve the functional outcomes of CSVD is still unknown.

In this study, we adopted a renal hypertensive animal model, stroke-prone renovascular hypertensive rat (RHRsp), as an animal model of CSVD. The RHRsp model was established using a two-kidney, two-clip(2k2c) procedure [16]. After the operation, the RHRsp presented with persistent hypertension. Studies have shown that RHRsp rats presented cerebral small artery remodeling with fibrinoid necrosis, hyalinosis, and apparent luminal narrowing [17]. Behavioral tests have also confirmed cognitive impairment in RHRsp [18]. These results suggest that RHRsp is a relatively ideal animal model of CSVD, particularly for the study of vascular pathological changes in hypertension-associated CSVD.

With this background, this study aimed to evaluate the role of EP3 receptors in CSVD, including (i) whether the deletion of EP3 attenuates vascular remodeling in RHRsp and (ii) whether the deletion of EP3 could further improve the function of animals, including changes in CBF and cognitive function.

## MATERIALS AND METHODS

### Animals and Treatment

All animal use procedures were strictly performed as per the Provision and General Recommendation of Chinese Experimental Animals Administration Legislation and were approved by the Fudan University Experimental Animal Science Department Animal Welfare and Ethics Review Board. Owing to the possible effect of sex hormones on hypertension induced by the two-kidney, two-clip (2k2c) procedure, experiments were performed in male rats. Animals were housed in rooms under controlled temperature (21±2°C), humidity (60±10%), and a 12 h light/dark cycle.

*EP3^-/-^*( *EP3* knockout) rats in a Sprague-Dawley (SD) background were created by Beijing Biocytogen (Beijing, China) using CRISPR/Cas9-based technology (validation of *EP3^-/-^* rats are provided in the Supplementary Material). Wild-type (WT) littermates (*EP3^+/+^* rats) were generated as experimental controls from EP3 receptor heterozygous mating. We used 30 *EP3^-/-^* and 30 WT rats.

Rats were subjected to either a 2k2c procedure to establish the RHRsp model or sham surgery for the control group. The establishment of the RHRsp model was performed as a 2k2c procedure, according to previous reports [16]. In brief, rats weighing 80-100 g were deeply anesthetized with sodium pentobarbital, and a median longitudinal incision was made on the abdominal skin of the rats. Both renal arteries of rats were exposed and placed with ring-shaped silver clips (provided by the Laboratory of Neurology, The First Affiliated Hospital of Sun Yat-sen University, China). Rats in the sham-operated group underwent the same procedure without clip placement.

Investigators responsible for functional assessment, outcome measurement, and data analysis were blinded to the experimental groups.

### EP3 KO rat generation

*EP3^-/-^*(knockout) rats on a Sprague-Dawley background were created by Beijing Biocytogen (Beijing, China) using CRISPR/Cas9-based technology. Briefly, single guide RNAs (sgRNAs) targeting the non-conservative region upstream of Exon1 and the non-conservative region upstream of Intron1 were transcribed in vitro and validated. Two active sgRNAs targeting 5-AGGTTCTTACATGGCAACAGTGG-3 and 5-GCAG CCCCTAG, GAAGCCGGTAGG-3 were selected. Then, the Cas9/sgRNAs were microinjected into the pronuclei of fertilized eggs from Sprague Dawley rats. The resulting live births were screened for deletions by PCR and Sanger sequencing. The selected founders were backcrossed to WT rats to generate a heterozygous F1 generation. The animals were genotyped by PCR using DNA isolated from tail samples with two pairs of primers (WT-F 5AATT GTCCAGGAGGGCAG-TCAAGTG-3 and Mut-R 5′ATTCCCTCCATAAGGATGGGGGTGG -3′; WT-F 5AATT- GTCCAGGAGGGCAGTCAAGTG-3 and WT- R 5′ AGTGGAGGCTGAAATCAAAGGA- GCA). WT littermates were generated as experimental controls from EP3 receptor heterozygous mating.

### Blood Pressure (BP) monitoring

BP measurements were performed in adult *EP3^-/-^* and WT rats aged 12 weeks. Two months and four months after the 2k2c procedure or sham operation, the BP of the animals was also measured. Awake BP measurement using the tail-cuff method (BP-98A; Softron Co, Tokyo, Japan): the day before BP was measured, animals were induced into the holding cage for 15 min to help the animals adapt to the environment. On the day of measurement, the instrument was preheated for 15 min before use, and then the rats were induced into the holding cage, with its tail being properly placed into the pressurized sensor sleeve. BP was measured only after the animals were calmed in the cage. The measurement was repeated three times at intervals greater than 5 min, and the average of each animal was taken. Unconscious BP measurement using invasive carotid artery catheterization: The rats were anesthetized with sodium pentobarbital and placed on a temperature-controlled pad. One side of the carotid artery was exposed, and a polyethylene tube prefilled with heparinized normal saline was inserted into the artery. BP was measured using a pressure transducer and recorded in real-time.

### Evaluation of CBF and Cognitive Function

Magnetic Resonance Imaging (MRI) with a dynamic susceptibility contrast perfusion-weighted imaging (DSC-PWI) was conducted to evaluate CBF of animals. MRI was performed using a 3.0-T MR scanner (Discovery MR 750, GE Healthcare, Milwaukee, WI, USA) with an animal phased-array coil. The animals were anesthetized with sodium pentobarbital and scanned in the prone position. A triplanar scout scan was performed first, followed by T2 weighted image (T2WI) and DSC-PWI. T2WI employed a fast spin-echo sequence with T2-weighted images: fast spin echo, repetition time/echo time (TE) = 4804/85 ms, slice thickness = 1.8 mm, spacing = 0.2 mm, Frequency field of view (FOV) = 6.0, phase FOV=0.8, and flip angle = 111°. DSC-PWI employed an echo-planar imaging sequence with TE = 17.8 ms, slice thickness = 2 mm, spacing = 0.2 mm, FOV = 6.0 mm, phase FOV = 0.8, and flip angle = 60°. Gadobutrol (Bayer HealthCare, Berlin, Germany) was injected at a dose of 1.5 mL/kg. After MRI scanning, all data were transmitted to the AW4.6 workstation (GE Healthcare) to acquire CBF images. All T2WI and CBF images were converted to NIfTI format using the dcm2nii program (http://nitrc.org/projects/dcm2nii) and analyzed using MRIcron software (www.nitrc.org/projects/mricron/). The volume of interest of each rat was drawn, covered the whole brain parenchyma in the T2WI, and saved as a mask. Then, the mask was overlaid onto the CBF map to acquire the readings of the CBF of the whole brain.

The Morris Water Maze test was conducted 16 weeks after the 2k2c operation to evaluate the cognitive function of the animals. The test was performed in a circular water pool, which was divided into four quadrants with four start positions. A transparent platform was hidden 1 cm below the surface of the water in one of the quadrants. After habituation in the pool without the platform for 2 min on day 0, the platform acquisition test was performed four times per day over the next 5 days from day 1 to day 5. The rats were released into the water at the four positions sequentially on each day and allowed to swim to search for the platform within 60 s. Time spent by the rat to find the hidden platform was recorded as the escape latency time. If the rat failed to find the platform within 60 s, it was gently guided onto the platform, and the escape latency time was recorded as 60 s. The rats were allowed to stay on the platform for 15 s each time. The platform was removed on day 6. The rats were allowed to swim for 60 s. The number of times crossing the former platform location and the time spent in the former platform quadrant of the rats in 60 s were recorded. All procedures were monitored using a video tracking system (EthoVision10.0, Noldus Information Technology Co., Ltd. Beijing).

### Brain Tissue Preparation

The rats were sacrificed 4-5 months after surgery and transcardially perfused with approximately 300 mL of 0.9% saline, followed by 200 mL of 4% paraformaldehyde (PFA) in 0.1 M phosphate-buffered saline (PBS, pH 7.4) within 5 min. The brain tissues were post-fixed in 4%PFA for 4-6 h and stored in 30% sucrose in 0.1 M PBS (pH 7.4) until the brain tissues fell to the bottom of the solution. The brains were sectioned into coronal serial slices (10 μm thickness) from approximately +5 mm bregma to -5 mm bregma.

### Immunofluorescence

The cryostat sections were treated with 0.25% Triton X-100 at room temperature (RT, 22-25°C) for 10 min. After washing three times in PBS, the sections were blocked with 1% bovine serum albumin for 1 h at RT. The sections were then incubated with primary antibodies overnight at 4°C, including EP3 (Sigma), αSMA (Sigma), collagen I (Abcam), collagen IV (Abcam), fibronectin (Abcam), and laminin (Abcam). The slides were then washed three times in 0.01 M PBS and incubated with Alexa Fluor Cy3- and 488-conjugated secondary antibodies (1:1000; Invitrogen Carlsbad, CA, USA) for 1 h at RT. Following an additional three washes in 0.01 M PBS, the slides were counterstained with DAPI. Finally, the sections were sealed with the anti-fade reagent.

The slides were photographed under a fluorescence microscope (Olympus BX-51, Tokyo, Japan). For each primary antibody, three sections were stained per animal. We divided each cross-section of the brain into three parts according to a previous study [19]: cortical gray matter, white matter, and deep gray matter. At least three random fields of each part were selected and photographed for further analysis. The cerebral small arteries (10-65 μm) were indicated by immunostaining for αSMA [18].

The fluorescence intensity of target proteins in cerebral small arteries in each field was calculated using ImageJ (1.53c) software, blinded to the groups. The final fluorescence intensity was determined using the mean value of all fields.

### Cell culture

Primary rat brain microvascular smooth muscle cells (BMVSMCs) were cultured from male 5-day-old rats by modification of a previously described procedure [20, 21]. Briefly, rat brains devoid of the cerebellum were placed in cold Hank’s balanced salt solution without Ca^2+^ and Mg^2+^ (D-Hanks). The pial membranes were removed, and the cerebral cortices were cleaned of white matter, scissor-minced, and homogenized in D-Hanks through a 70-µm nylon mesh. The microvessel pellet was collected from above the filter, centrifuged at 3000 rpm for 8 min, and then resuspended with 1.0 mg/mL collagenase (CLS type I; Worthington, Freehold) at 37°C for 5-10 min with periodic shaking. After digestion, the microvessel pellet was collected by centrifugation at 3000 rpm for 8 min and cultured in Dulbecco’s Modified Eagle Medium (Hyclone, Logan, UT) supplemented with 100 U/mL penicillin, 0.1 mg/mL streptomycin, 2 mM L-glutamine, and 20% FBS in a humidified incubator (Thermo Scientific) at 37°C in 5% CO_2_/95% air. After 1 week, BMVSMCs were transferred into new cell culture flasks. Cultured BMVSMCs were used after passage 3 with a vascular smooth muscle cells (VSMCs) purity of over 95%. Immunocytochemistry anti-αSMA was used for the identification of VSMCs.

Before stimulation, rat BMVSMCs were growth-arrested by incubation in a serum-free medium for 24 h. Angiotensin II (ANGII, Sigma, 1 µmol/L, 24 h), LY364947 (Selleck, 3.15 µmol/L, 24 h), and L798106 (Cayman, 10 µmol/L, 24 h) were used for stimulation.

### Western blotting

The concentration of proteins extracted from rat BMVSMCs was determined using a Pierce BCA Protein Assay Kit (Pierce, Rockford, IL, USA). Equal quantities of proteins were denatured and resolved by 8%-10% SDS-PAGE, transferred to nitrocellulose membranes, incubated with 5% skimmed milk for 1 h, and then incubated with primary antibodies overnight at 4°C. The membranes were then conjugated with a horseradish peroxidase-labeled secondary antibody for 1 h at room temperature. Blots were developed using an enhanced chemiluminescence reagent (Thermo Scientific), followed by densitometric quantification using ImageJ (1.53 c).

### RNA extraction and real-time PCR

Total RNA from the brains of animals was extracted using TRIzol reagent (Invitrogen), according to the manufacturer’s protocols. Briefly, total RNA (2 µg) was reverse-transcribed to cDNA using the RevertAid First Strand cDNA Synthesis Kit (Thermo), according to the manufacturer’s instructions. The resulting cDNA was amplified using 40 PCR cycles by real-time PCR. Each sample was analyzed in triplicates and normalized to a reference RNA within the sample. The primer sequences used for PCR are summarized in Table 1.

### Statistical Analysis

Prism software (version 8.0) was used for data analysis. Data are presented as the mean ± standard deviation. All data sets were normally distributed, and the variance was homogeneous among all groups, as determined by one-way analysis of variance. Differences between two groups were analyzed using a t-test, and data from multiple groups were compared using a one-way analysis of variance. Statistical significance was set at p<0.05.

## RESULTS

### EP3 is upregulated in cerebral small arteries of RHRsp, and deletion of EP3 does not affect blood pressure

**Figure 1.**
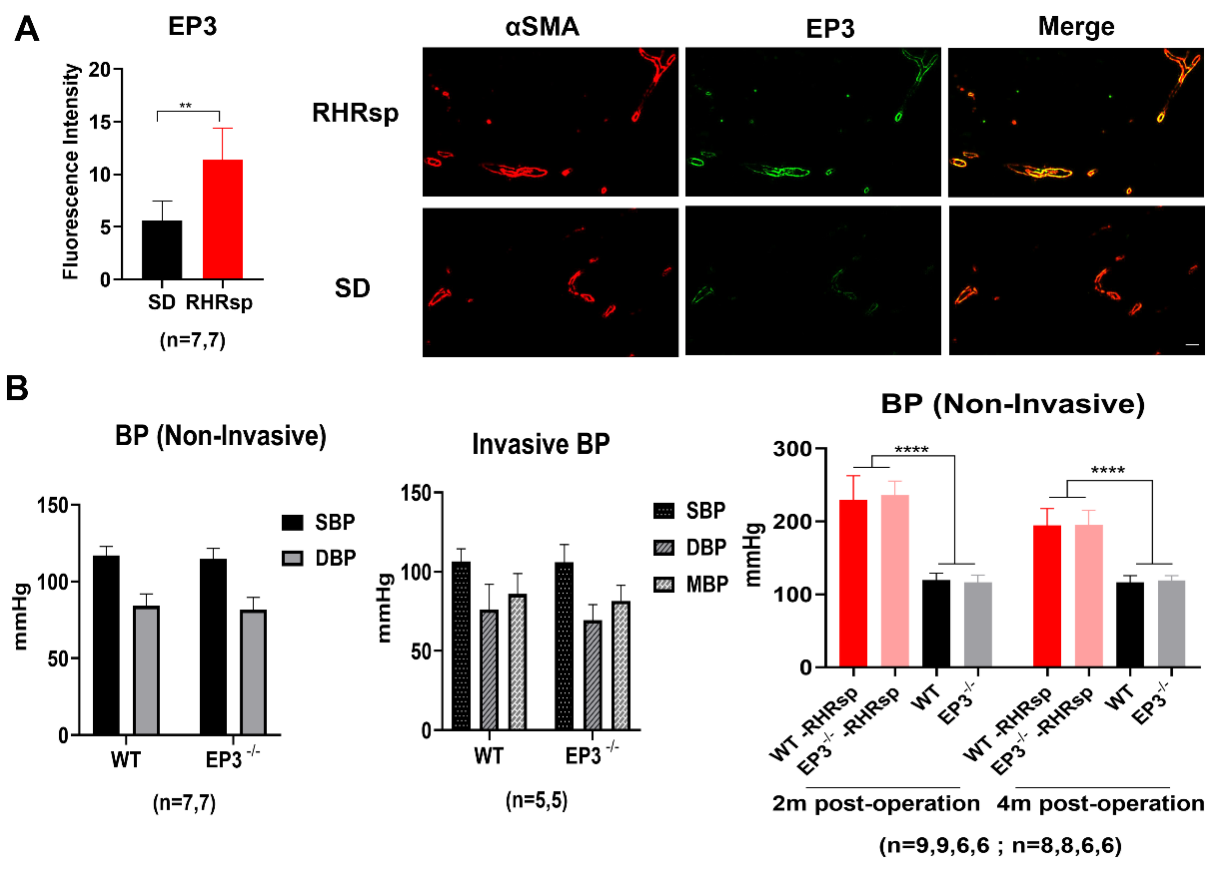
EP3 is upregulated in cerebral small arteries of RHRsp and deletion of *EP3* does not affect BP. (A) Immunofluorescence for EP3 (Green) and αSMA (Red) of SD and RHRsp. The cerebral small arteries (10-65 μm) are indicated by the immunostaining of αSMA. Bar, 50 µm. (B) Blood pressure in SD and RHRsp. EP3, E prostanoid 3; SD, Sprague Dawley rat; RHRsp, Stroke-prone renovascular hypertensive rat; αSMA, smooth muscle actin alpha; BP, blood pressure; SBP, systolic blood pressure; DBP, diastolic blood pressure; MBP, mean blood pressure; m, month; **, p<0.01; ****, p<0.0001.

COX-2/ PGE2 signaling is an important inflammatory pathway. Hypertension is strongly associated with the augmented expression of the COX-2/ PGE_2_/EP3 axis in both human and animal models [13, 14]. We also observed significantly elevated expression of EP3 in the cerebral small arteries of RHRsp by immunofluorescence (Fig. 1A), indicating that the PGE2/EP3 axis might be involved in the progression of CSVD induced by hypertension.

We then constructed the RHRsp model in *EP3* knockout rats (*EP3^-/-^*) to determine whether EP3 receptor deficiency can attenuate the development of CSVD. First, we sought to determine whether the deletion of *EP3* can affect the BP of the animals as hypertension is a critical risk factor for CSVD, and PGE_2_ signaling is reported to have major effects on BP control through its receptors [22]. We used two methods to monitor BP, including conscious BP measurement using the tail-cuff method and unconscious BP measurement using invasive carotid artery catheterization. The results showed no significant difference in the baseline BP of either group (Fig. 1B). Furthermore, we measured the BP of rats after the 2k2c procedure. Both *EP3^-/-^* and WT rats after the 2k2c procedure showed significantly higher BP compared to the sham-operated groups. However, there was still no difference between *EP3^-/-^* rats and WT rats within the hypertensive group (Fig. 1B), indicating that *EP3* knockout did not affect both the baseline BP and induction of hypertension by the 2k2c procedure.

### Deletion of EP3 attenuates the overexpression of ECM in the cerebral small arteries of RHRsp

**Figure 2.**
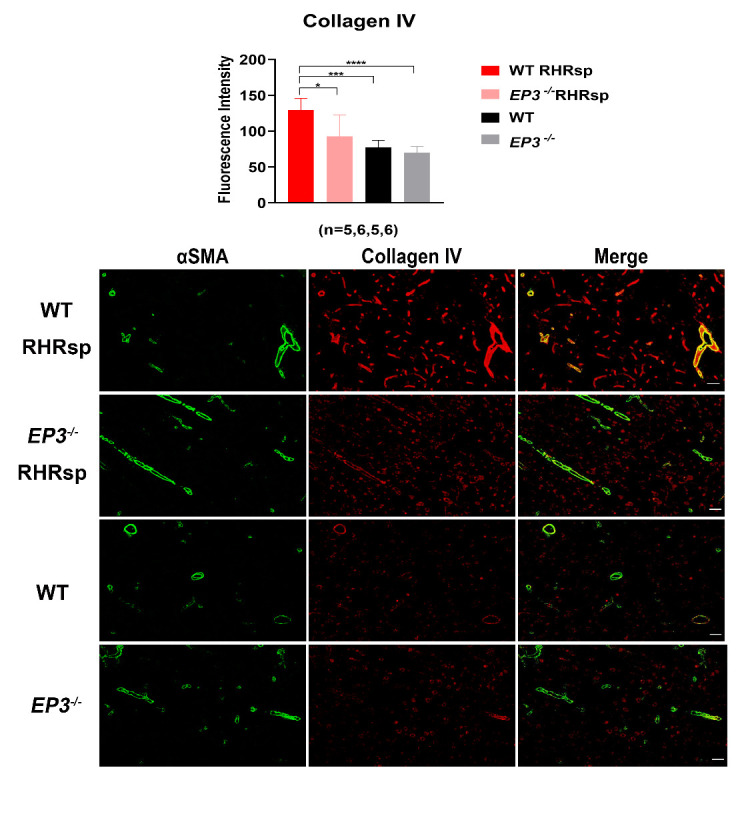
The deposition of collagen IV in the vessel wall of cerebral small arteries. Immunofluorescence for αSMA (Green) and collagen IV (Red) of the animals. The cerebral small arteries (10-65 μm) are indicated by the immunostaining of αSMA. Bar, 50 µm. EP3, E prostanoid 3; RHRsp, Stroke-prone renovascular hypertensive rat; αSMA, smooth muscle actin alpha, *p<0.05, ***p<0.001, ****p<0.0001.

**Figure 3.**
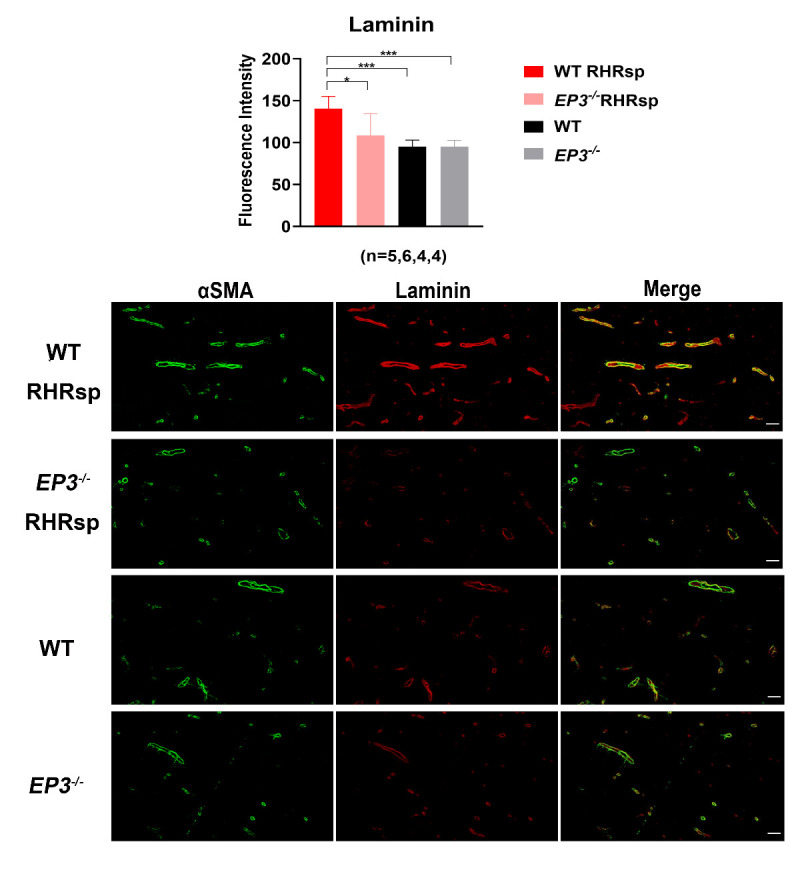
The deposition of laminin in the vessel wall of cerebral small arteries. Immunofluorescence for αSMA (Green) and laminin (Red) of the animals. The cerebral small arteries (10-65 μm) are indicated by the immunostaining of αSMA. Bar, 50 µm. EP3, E prostanoid 3; RHRsp, Stroke-prone renovascular hypertensive rat; αSMA, smooth muscle actin alpha, *p<0.05, ***p<0.001.

**Figure 4.**
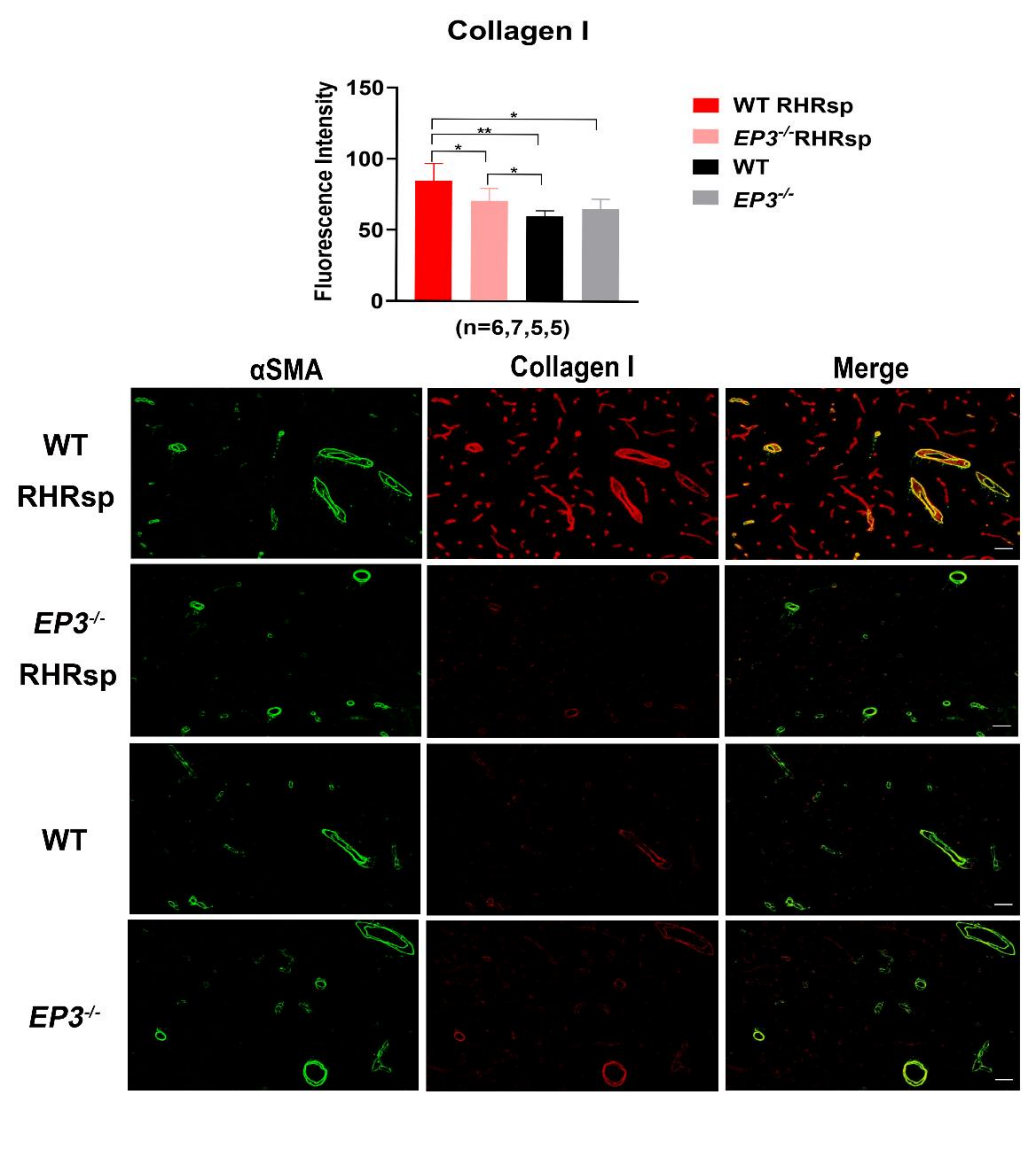
The deposition of collagen I in the vessel wall of cerebral small arteries. Immunofluorescence for αSMA (Green) and collagen I (Red) of the animals. The cerebral small arteries (10-65 μm) are indicated by the immunostaining of αSMA. Bar, 50 µm. EP3, E prostanoid 3; RHRsp, Stroke-prone renovascular hypertensive rat; αSMA, smooth muscle actin alpha, *p<0.05, **p<0.01.

**Figure 5.**
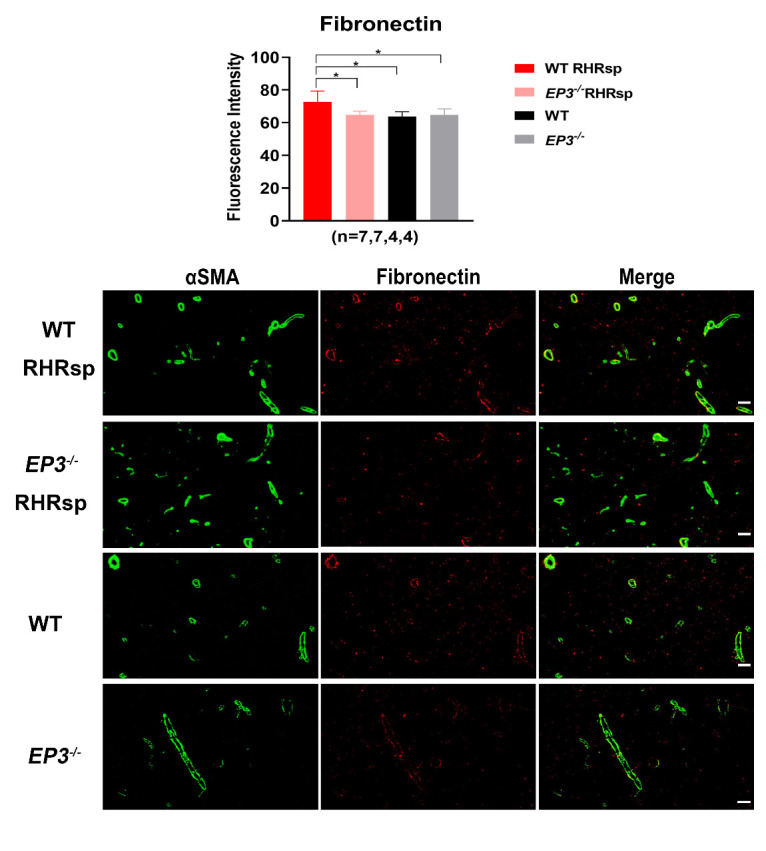
The deposition of fibronectin in the vessel wall of cerebral small arteries. Immunofluorescence for αSMA (Green) and fibronectin (Red) of the animals. The cerebral small arteries (10-65 μm) are indicated by the immunostaining of αSMA. Bar, 50 µm. EP3, E prostanoid 3; RHRsp, Stroke-prone renovascular hypertensive rat; αSMA, smooth muscle actin alpha, *p<0.05.

Next, we tested whether the deletion of *EP3* modulates the progression of cerebral small artery remodeling in RHRsp. Two months after the 2k2c procedure, rats in the RHRsp group developed stable hypertension. By the end of 4-5 months, the cerebral small arteries of RHRsp showed a significantly higher expression of ECM, including fibronectin, laminin, collagen I, and collagen IV, compared with that observed in controls with normal BP. Intriguingly, *EP3^-/-^* RHRsp rats displayed a significant reduction in all four types of ECM, compared with that in WT RHRsp (Fig. 2, Fig. 3, Fig. 4 and Fig. 5). These results indicated that despite no effect on hypertension, the deletion of *EP3* still attenuated the overexpression of ECM in the cerebral small arteries of RHRsp.

### Deletion of EP3 decreases the expression of ECM in BMVSMCs under ANGII stimulation

**Figure 6.**
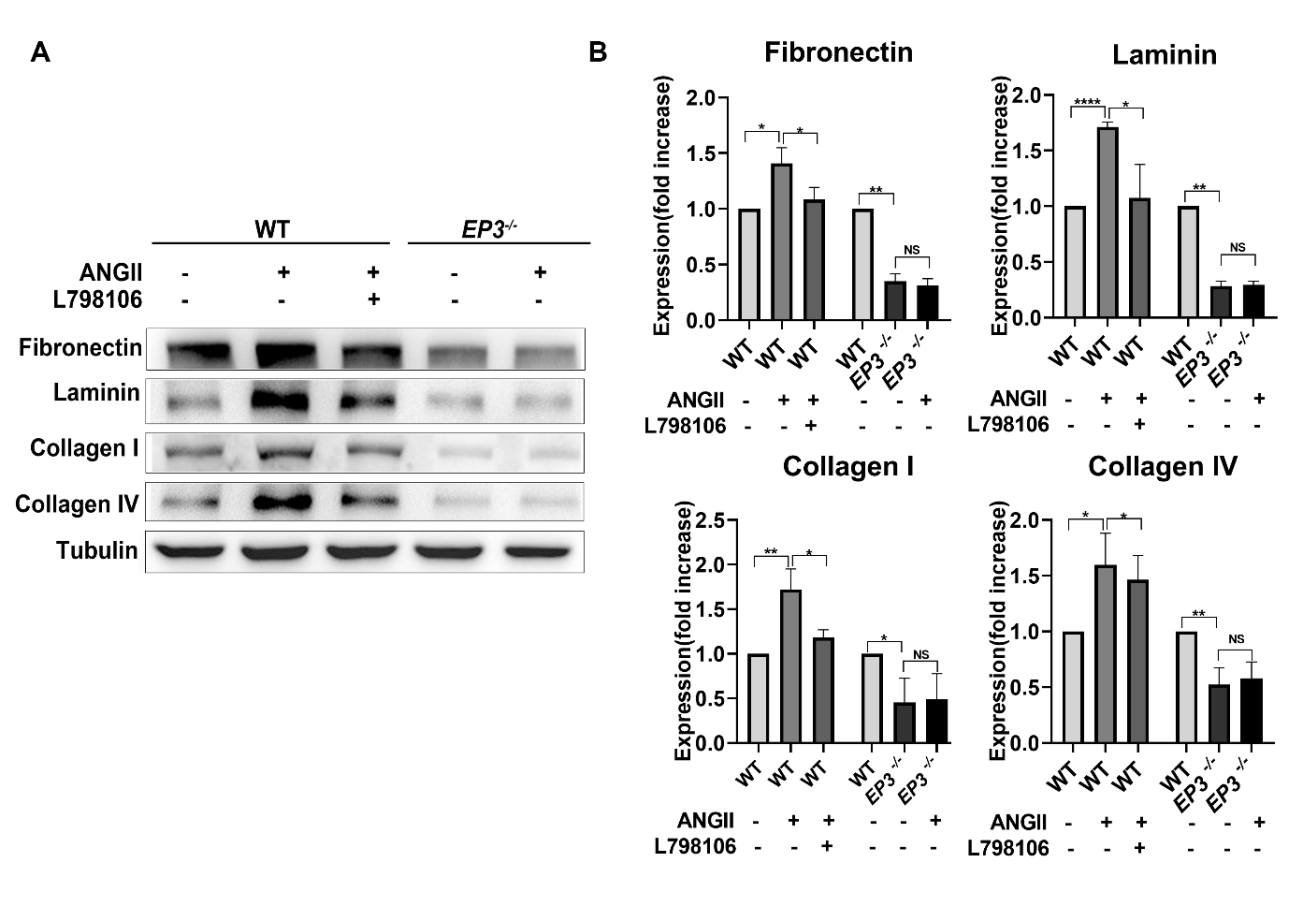
Change of extracellular matrix expression in rat BMVSMCs under different stimulation. (A) Representative western blots and quantification of the expression of fibronectin, laminin, collagen I, and collagen IV in rat BMVSMCs under different stimulation. (B) Quantification of western blots of fibronectin, laminin, collagen I, and collagen IV. EP3, E prostanoid 3; BMVSMCs, brain microvascular smooth muscle cells; ANGII, Angiotensin II. *, p<0.05; **, p<0.01; ****, p<0.0001.

As shown above, our results confirmed that deletion of *EP3* successfully attenuated cerebral artery remodeling in RHRsp without alleviating high blood pressure. Hence, we assumed that EP3 may directly regulate the expression of ECM by affecting VSMCs. To further study the mechanism by which EP3 regulates VSMCs to express ECM in CSVD, BMVSMCs were extracted from both WT and *EP3^-/-^* rats. Previous studies have shown that in vitro VSMCs exposed to ANG II display increased levels of ECM, and in vivo infusion of ANG II increases the expression of vascular ECM in animals [23]. Furthermore, both hypertensive humans and animals, including RHRsp, were reported to have elevated levels of ANGII [24, 25]. Hence, we used ANGII to stimulate BMVSMCs to induce elevated expression of ECM in vitro. As shown in Fig. 6, upon stimulation with ANGII, BMVSMCs exhibited significantly higher expression of ECM proteins, including fibronectin, laminin, collagen I, and collagen IV. We found that the addition of the EP3 inhibitor L798106 significantly alleviated this increase in ECM expression induced by ANGII. Furthermore, the deletion of *EP3* genes can significantly inhibit the baseline expression of ECM (without ANGII stimulation). Even under the stimulation of ANGII, a significantly higher expression of ECM was not observed in *EP3^-/-^* rat BMVSMCs. These results suggest that *EP3* gene knockout can reverse the ANGII-induced increase in ECM expression in rat BMVSMCs.

### Deletion of EP3 decreases the expression of ECM by suppressing TGF-β1 signaling in BMVSMCs under ANGII stimulation

**Figure 7.**
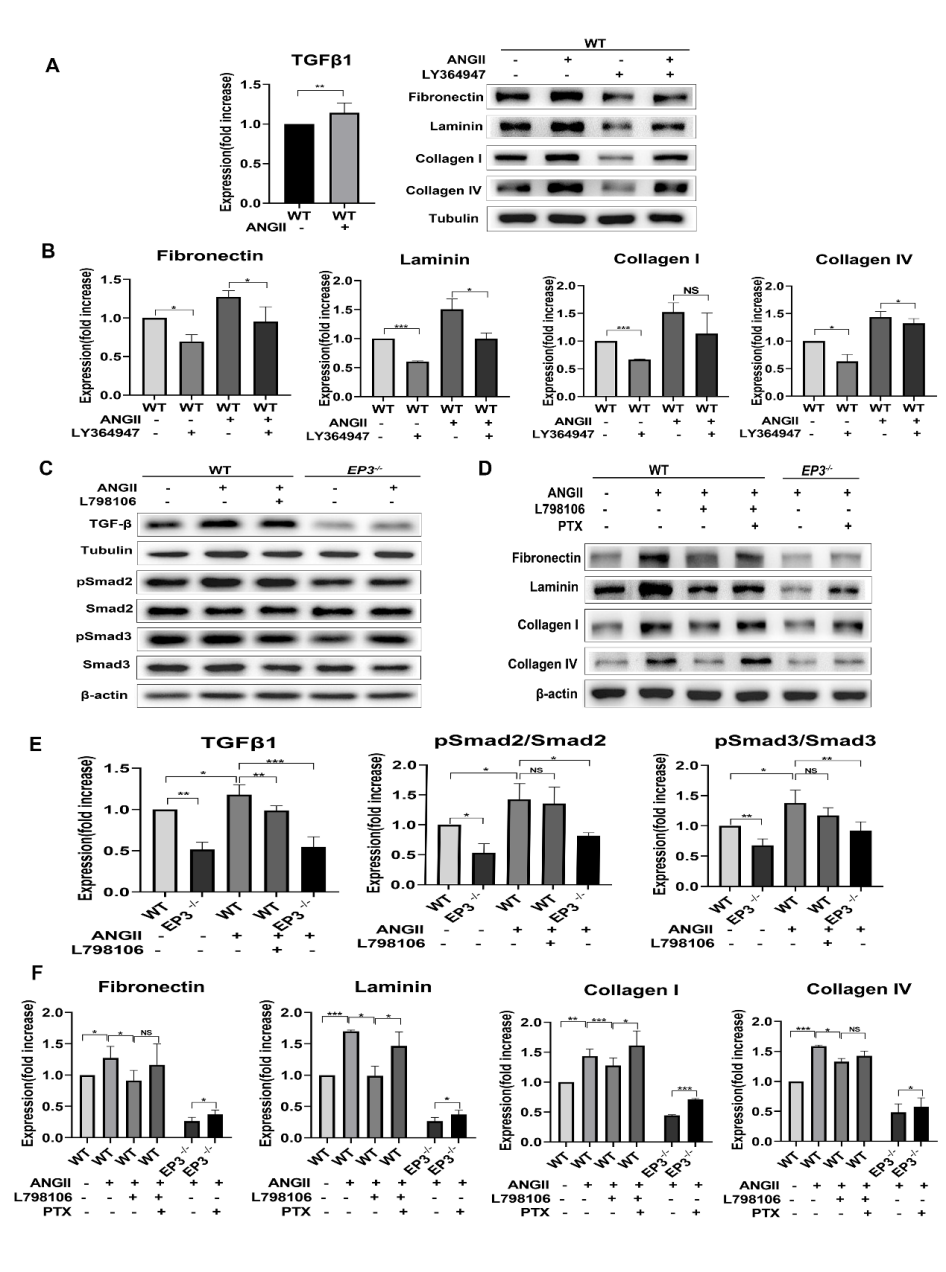
Quantification of western analysis. (A-B) TGF-β1 is elevated in rat BMVSMCs under ANGII stimulation. Suppressing TGF-β1 by LY364947 reverses the high expression of ECM in rat BMVSMCs under ANGII stimulation. (C, E) Deletion of *EP3* rescues ANGII-induced activation of TGF-β1/ SMAD2/signaling. (D, F) The downregulation of ECM by the inhibition of EP3 is abolished by pretreatment with PTX in BMVSMCs. EP3, E prostanoid 3; BMVSMCs, brain microvascular smooth muscle cells; ECM, extracellular matrix; TGF-β1, transforming growth factor-beta1; ANGII,Angiotensin II. *, p<0.05; **, p<0.01; ***, p<0.001; NS, no significant statistical difference.

TGF-β1 signaling has long been reported to play a role in regulating ECM expression [26]. It has also been suggested that the knockout of *EP3* downregulates the expression of ECM in pulmonary arterioles by suppressing the TGF-β1/Smad pathway [15]. In this study, we sought to determine whether EP3 regulates the expression of ECM by altering TGF-β1 signaling. As shown in Fig. 7, western blot analyses confirmed that the level of TGF-β1 and phosphorylation of Smad2/3 were elevated in rat BMVSMCs following ANGII stimulation. LY364947 is a compound described as selectively inhibiting TGF-β1 receptor kinase activity [27, 28]. Administration of LY364947 attenuated the high expression of ECM induced by ANGII in rat BMVSMCs (Fig. 7A-B). Furthermore, the deletion of *EP3* down-regulated ANGII-induced activation of TGF-β1 signaling, as evidenced by a decreased level of TGF-β1 and phosphorylation of Smad2/3 (Fig. 7C and E). These results indicated that suppressing TGF-β1 signaling could be one of the possible pathways through which deletion of *EP3* decreases the expression of ECM in BMVSMCs under ANGII stimulation.

Previous studies have shown that the EP3 receptor modulates multiple intracellular signaling pathways by coupling different types of heterotrimeric G proteins [29]. As shown in Fig. 7D and Fig. 7F, the downregulation of ECM by EP3 inhibition was abolished by pretreatment with pertussis toxin (PTX) in BMVSMCs, indicating that EP3 signaling regulated the expression of ECM via the PTX-sensitive G protein Gi/o in BMVSMCs.

### Deletion of EP3 attenuates decreased CBF in RHRsp

**Figure 8.**
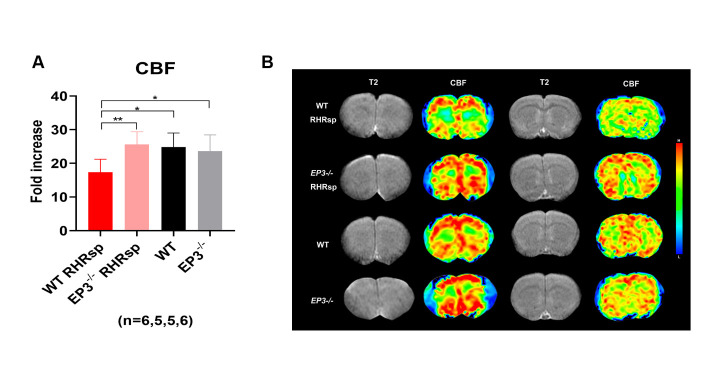
Deletion of *EP3* attenuates reduced CBF in RHRsp. (A) Quantitative results of CBF. (B) Representative picture of CBF. EP3, E prostanoid 3; CBF, cerebral blood flow; RHRsp, Stroke-prone renovascular hypertensive rat; H, high; L, low; *, p<0.05, **, p<0.01.

Cerebral small artery remodeling is the leading cause of decreased CBF in patients [30]. To determine whether attenuated vascular remodeling caused by *EP3* deletion can further improve CBF in RHRsp, we used PWI to monitor the global CBF of the animals. T2-weighted images and representative CBF images of the animals are shown in Fig. 8. Consistent with previous studies on *EP3^-/-^* mice [31] or mice treated with selective antagonists of EP3 [32], our results showed no difference in CBF between normal WT rats and *EP3^-/-^* rats. As decreased CBF is observed in patients with CSVD [33], our results showed that RHRsp demonstrated a lower global CBF compared to the control rats. Intriguingly, *EP3^-/-^* RHRsp rats displayed a recovery of global CBF compared to WT RHRsp. These results indicate that the deletion of *EP3* can improve CBF in RHRsp, confirming our hypothesis that the deletion of *EP3* can not only reverse vascular structure changes but also contribute to the functional recovery of blood supply in the brain.

### Deletion of EP3 attenuates cognitive impairment in RHRsp

**Figure 9.**
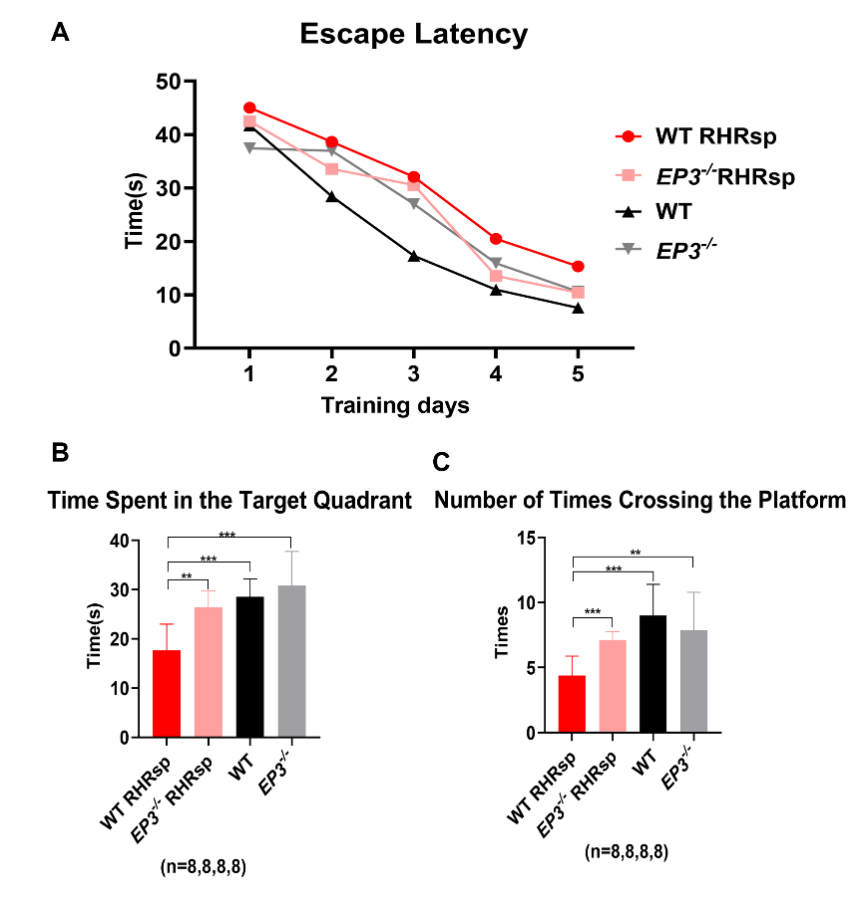
Deletion of *EP3* attenuates cognitive impairment in RHRsp. (A) Morris water maze test: escape latency in place navigation test. (B) Morris water maze test: time spent in the target quadrant in the spatial probe test. (C) Morris water maze test: the number of times crossing the platform in the spatial probe test. EP3, E prostanoid 3; RHRsp, Stroke-prone renovascular hypertensive rat; **, p<0.01; ***, p<0.001.

As seen above, deletion of *EP3* successfully attenuated vascular remodeling in RHRsp and improved the cerebral blood supply of the animals. We assume that these improvements may also help attenuate cognitive impairment in RHRsp. To this end, we used the Morris water maze test to evaluate the cognitive function of the animals. The Morris water maze test was designed to assess spatial learning and memory ability. During the hidden platform acquisition phase, on average, rats in all groups showed a progressive decrease in escape latency, indicating that subjects could learn the location of the platform. However, compared with the sham-operated group, RHRsp rats had a longer escape latency, and a statistical difference was observed on the third and fifth days. Deletion of *EP3* (*EP3^-/-^* RHRsp) shortened the escape latency. However, this difference was not statistically significant (Fig. 9). A no-platform probe test was conducted 24 h after the final day 5 training trial. Figure 9 shows that the time spent in the target quadrant was lower in RHRsp rats than in sham-operated rats. Deletion of *EP3* (*EP3^-/-^* RHRsp) reversed this effect. The average number of times crossing the platform was higher in RHRsp than in sham-operated rats, and the deletion of *EP3* (*EP3^-/-^* RHRsp) significantly improved the number. Collectively, these results show damaged spatial learning and memory ability in RHRsp, and the deletion of *EP3* in RHRsp can rescue cognitive impairment. We cautiously speculate that the improvement of cognitive function in *EP3^-/-^* RHRsp could be partially attributed to the reduced vascular remodeling and increased CBF by the deletion of *EP3*.

## DISCUSSION

In this study, we first observed increased EP3 expression in the cerebral small arteries of an animal model of CSVD. We demonstrated that *EP3* gene knockout can reverse cerebral small artery remodeling, possibly by downregulating TGF-β1 signaling. Furthermore, we found that vascular structure improvement by *EP3* deletion also attenuated the decreased CBF of RHRsp and eventually yielded success in reducing the cognitive impairment of the animals.

Increased COX-2/PGE_2_/EP3 signaling has long been observed in hypertensive patients and animals, and inhibition of the signaling pathway attenuates vascular remodeling in hypertension [13, 14]. Since COX-2/PGE_2_/EP3 signaling is found to affect BP [22], it is essential to know whether the improvement of vascular remodeling by inhibition of COX-2/PGE_2_/EP3 signaling in vivo is accompanied by a decrease in hypertension. In this study, our results indicated that *EP3* knockout did not affect the baseline BP of the rats, which is consistent with research on *EP3^-/-^* mice [31, 34]. Although previous studies have shown that *EP3* gene knockout relieves hypertension in ANGII perfusion mice [35] and salt-induced hypertensive mice [34], our results suggest that *EP3* gene knockout did not affect the hypertensive response induced by the 2k2c operation in rats. Differences in hypertension induction methods and genetic backgrounds among studies may explain the different results [36]. The mechanism of persistent hypertension in RHRsp is much more complex. After the surgery, as juvenile rats grew, the renal arteries were gradually narrowed by the fixed-size silver clamps, gradually making the rats’ bilateral kidneys ischemic. Continuous renal ischemia activates the renin-angiotensin system and increases the release of sympathetic neurotransmitters, endothelin, aldosterone, and other substances; thereby, maintaining the persistence of hypertension [16, 25]. Our results indicated that EP3 deletion could improve vascular remodeling in CSVD, independent of hypertension control. These results also suggest that targeting COX-2/PGE2/EP3 signaling could be a promising treatment for CSVD in addition to regular antihypertensive medication.

Since the deletion of *EP3* attenuated ECM over-expression in the cerebral small arteries of *EP3^-/-^*RHRsp without relieving hypertension, we assumed that direct regulation of VSMCs by the EP3 gene could be critical in this process. Our in vitro study later confirmed that *EP3* can directly regulate the expression of ECM in VSMCs under stimulation by ANGII. A similar effect of EP3 on VSMCs has already been reported in pulmonary arteriolar smooth muscle cells [15], and research has also suggested that EP3 gene knockout can reduce the migration and polarity of VSMCs [37]. TGF-β1 signaling has long been found to regulate ECM production. Studies have indicated an elevated level of TGF-β1 in both patients with hypertension [38] and hypertensive animal models [39]. Our results demonstrated that the level of TGF-β1 and phosphorylation of Smad2/3 were elevated in rat BMVSMCs under ANGII stimulation and were relevant to the high expression of ECM. Deletion of *EP3* can reverse increased TGF-β1/Smad signaling and attenuate the high expression of ECM. Previous studies have also revealed that different receptors of PGE_2_ (EP1-4) participate in the regulation of ECM expression by affecting TGF-β1/Smad signaling. For example, studies have shown that the TGF-β1 pathway is involved in regulating the expression of renal vascular ECM, which is closely related to the occurrence of renal fibrosis. Activation of EP1 and EP3 receptors promotes renal fibrosis and accelerates renal damage [40], while activation of EP2 [41] and EP4 [42] receptors can inhibit TGF-β1-mediated increased expression of ECM in renal mesangial cells and alleviate renal damage. PGE2 can also inhibit TGF-β1 /Smad signaling via the EP2 receptor, thereby reducing the expression of various types of collagens as well as the formation of hypertrophic scars [43]. These results suggest that the role of the COX2/PGE_2_/EP axis in vascular remodeling in diseases is a complex issue. In different tissues and organs or under different pathological conditions, the COX2/PGE_2_ axis may regulate ECM expression through TGF-β1 signaling by binding to different receptors.

In our study, deletion of *EP3* further improved CBF and cognitive function in RHRsp. We assumed that this could be partially attributed to reduced vascular remodeling under EP3 deletion. However, our research cannot rule out other possible mechanisms of this process. PGE_2_ acts as a vasodilator and vasodepressor through four distinct EP receptors, and stimulation of EP3 triggers processes leading to vasoconstriction [44]. It has been reported that EP3 activation triggers vasoconstriction in porcine middle cerebral arteries [45]and the guinea pig aorta [46]. *EP3* deletion may have contributed to the improvement of CBF in RHRsp by reversing the enhanced vascular constriction under hypertension. Our study also showed an improved cognitive function accompanied by increased CBF in *EP3^-/-^* RHRsp. In addition to vascular protection, *EP3* deletion may preserve cognitive function in RHRsp through direct neuronal protection. Previous studies have indicated that blocking the EP3 receptor with L798106 rescued the expression of synaptic plasticity-related proteins in an animal model of surgery-induced memory deficits [47]. Although RHRsp is a relatively ideal animal model for the study of vascular remodeling in CSVD, this animal model does not present obvious white matter damage or cerebral microbleeds. Therefore, future studies using different animal models are needed to explore whether EP3 also affects other pathological lesions of CSVD.

Although EP3 antagonists are not widely used in clinical therapy, research has demonstrated that blockade of EP3 has been linked to numerous therapeutic areas, including the treatment of pain, diabetes, and cardiovascular disease, specifically thrombosis [48, 49]. Selective antagonists of the EP3 receptor include L798106, ONO-AE3-240, and DG-041 [49]. Preclinical and clinical evaluations were performed using these agents, especially DG-041. Single-dose and multiple-dose clinical trials with DG-041 suggested that the compound was well tolerated [50, 51]. Studies using DG-041 in healthy patients showed that DG-041 reduced platelet aggregation while preserving hemostatic function [51]. Further studies from bench to clinic are required.

In conclusion, our study revealed that the deletion of the *EP3* gene can significantly improve the over-expression of ECM in cerebral small arteries of CSVD rats, leading to improved CBF and cognitive function, and the effect may be partially through downregulation of TGF-β1 /Smad signaling. Thus, blockade of the EP3 receptor may be a promising strategy for the treatment of CSVD.

## Supplementary Materials

The Supplementary data can be found online at: www.aginganddisease.org/EN/10.14336/AD.2021.0627.


