# Supplementary material for: EP3 Receptor Deficiency Improves Vascular Remodeling and Cognitive Impairment in Cerebral Small Vessel Disease
Source: Aging Dis. 2022 Feb 1;13(1):313–28. doi: 10.14336/AD.2021.0627 (PMC8782563; doi:10.14336/AD.2021.0627)
Supplement: Supplementary file 1 [file AD-13-1-313-s.pdf]

SUPPLEMENTARY DATA

EP3 Receptor Deficiency Improves Vascular Remodeling and Cognitive Impairment in Cerebral Small Vessel Disease

Na Liu[1,#], Jie Tang[1,#], Yang Xue[1], Vincent Mok[2], Miaoyi Zhang[3], Xue Ren[1], Yilong Wang[4*], Jianhui Fu[1*]

© 2021. Liu N et al. Published online at http://www.aginganddisease.org/EN/10.14336/AD.2021.0627

SUPPLEMENTARY DATA

Supplementary Table 1. Primer sequences used for PCR

Gene	Primer sequence（5'-3')
GAPDH	CTGGAGAAACCTGCCAAGTATG<br>GGTGGAAGAATGGGAGTTGCT
EP3	CGCCGCTATTGATAATGATGCTG<br>CACTCCTTCTCCTTTCCCATCT

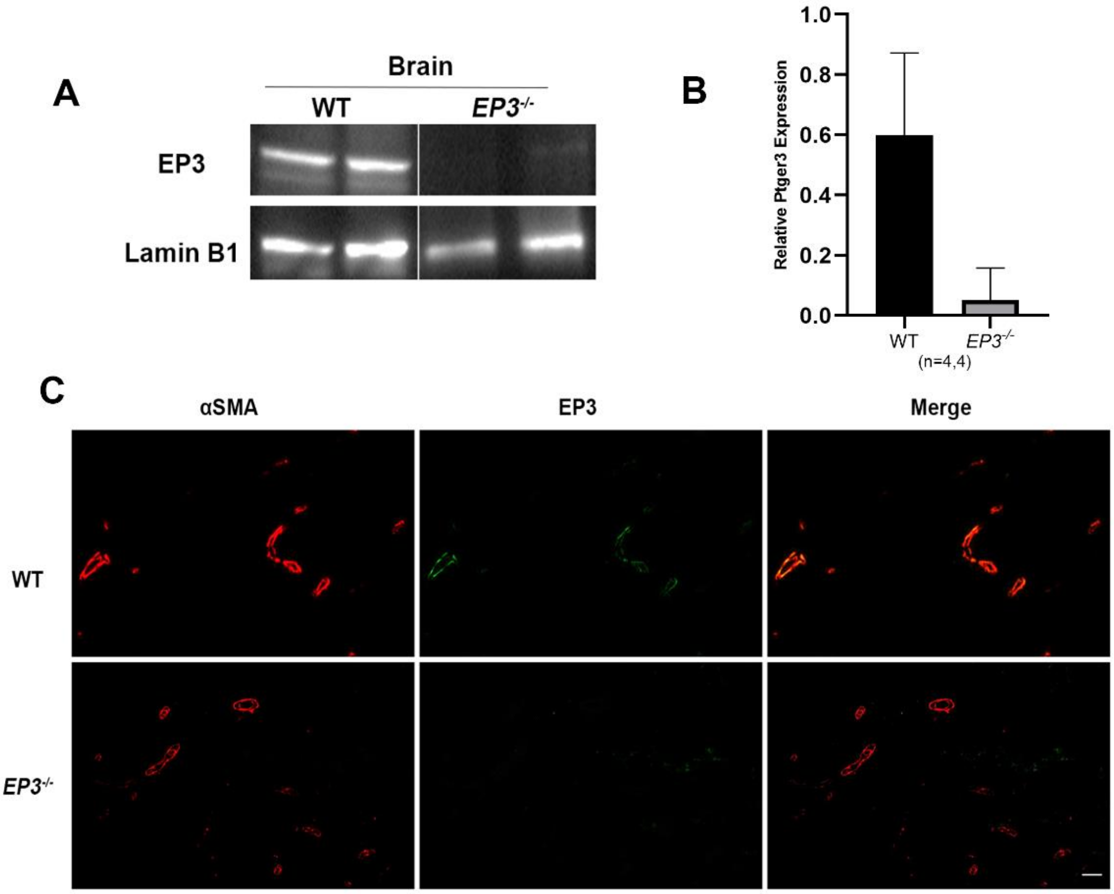

Supplementary Figure 1. Validation of *EP3*$^{-/-}$ rat. **(A)** Absence of EP3 protein in the brains of *EP3*$^{-/-}$ rats by western blotting. **(B)** Quantitative real-time (RT)-polymerase chain reaction (PCR) analysis of brain gene expression in WT and *EP3*$^{-/-}$ rats. **(C)** Absence of EP3 protein in the cerebral small arteries of *EP3*$^{-/-}$ rats as shown by immunofluorescence, αSMA (red), and EP3 (green). The cerebral small arteries (10–65 µm) are indicated by immunostaining for αSMA. Bar, 50 µm. EP3, E prostanoid 3; RHRsp, stroke-prone renovascular hypertensive rat; αSMA, smooth muscle actin alpha.

© 2021. Liu N et al. Published online at http://www.aginganddisease.org/EN/10.14336/AD.2021.0627

SUPPLEMENTARY DATA

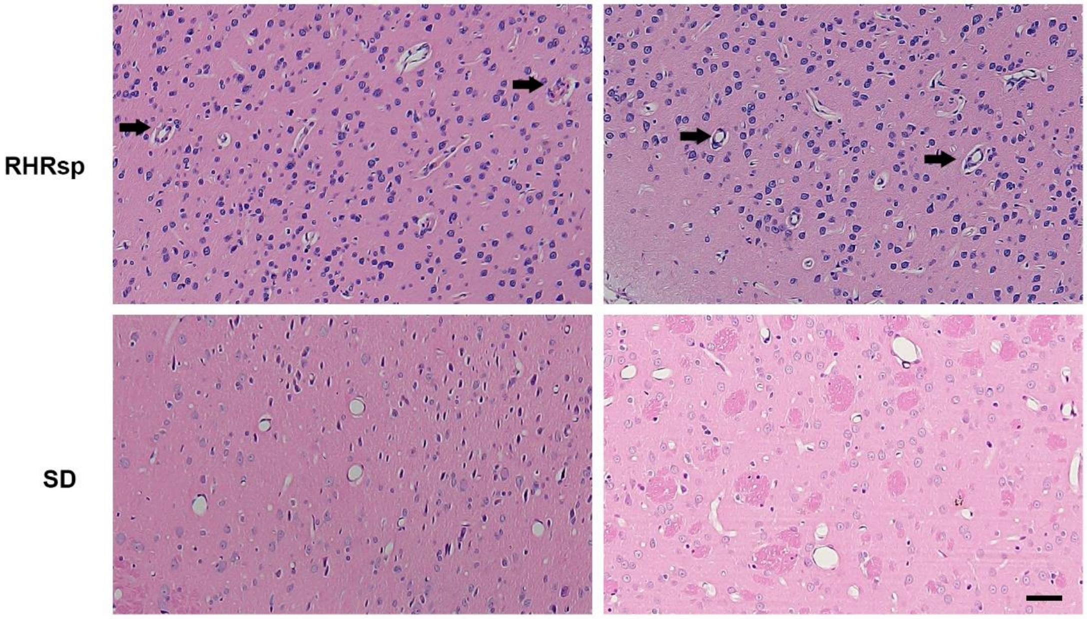

Supplementary Figure 2. Hematoxylin & Eosin (HE) staining of animals. RHRsp presented typical cerebral small vessel remodeling of cerebral small vessel disease (CSVD): thickening of the vessel wall, decrease in the inner diameter with enlarged perivascular space (indicated by black arrows). Bar, 50 µm. RHRsp, stroke-prone renovascular hypertensive rats; SD, Sprague-Dawley rats.

© 2021. Liu N et al. Published online at http://www.aginganddisease.org/EN/10.14336/AD.2021.0627
