# Supplementary figures and images for: EP3 Receptor Deficiency Improves Vascular Remodeling and Cognitive Impairment in Cerebral Small Vessel Disease
Source: Aging Dis. 2022 Feb 1;13(1):313–28. doi: 10.14336/AD.2021.0627 (PMC8782563; doi:10.14336/AD.2021.0627)

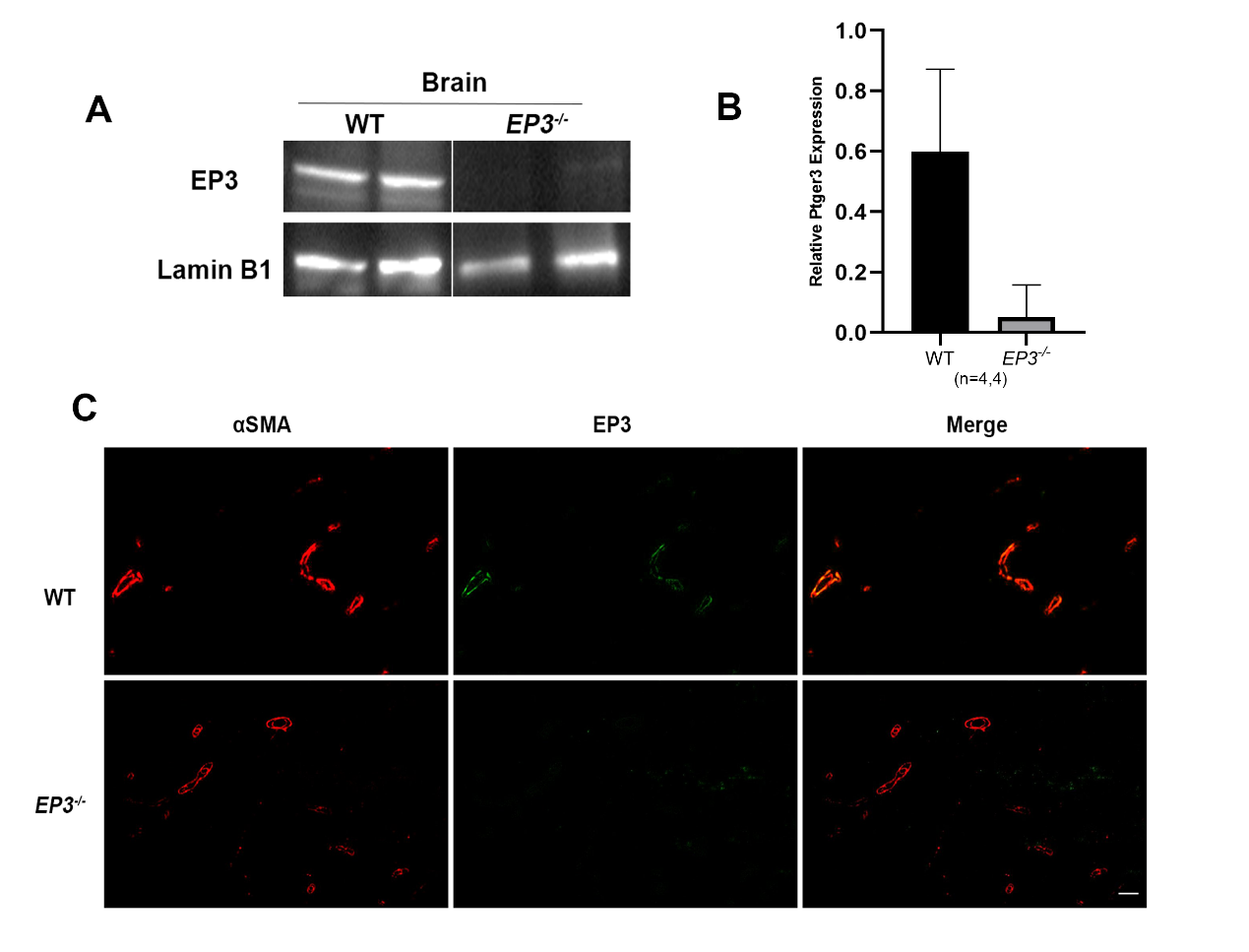

Supplement: Supplementary file 2 [file ad-13-1-313-s-g1.tif]

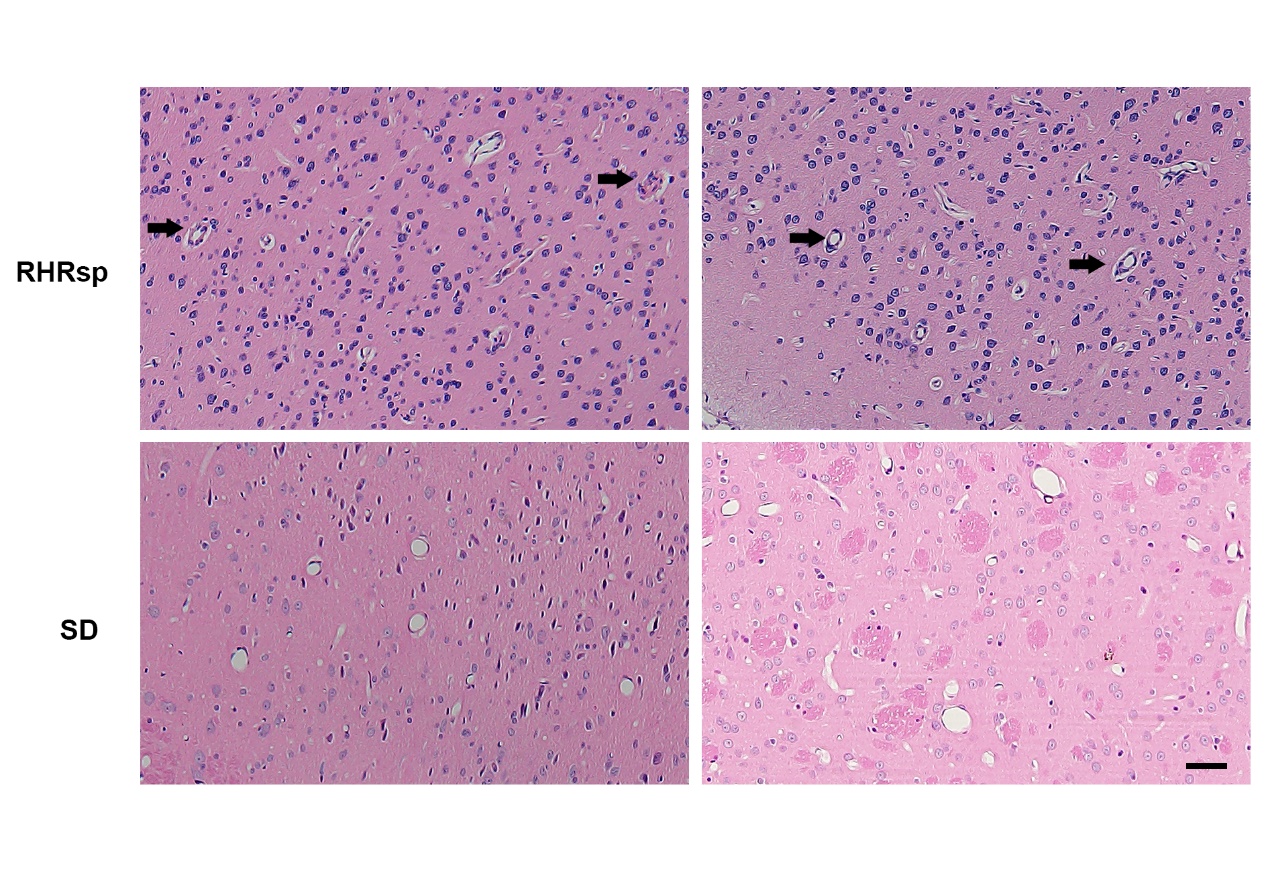

Supplement: Supplementary file 3 [file ad-13-1-313-s-g2.jpg]
